# Supplementary figures and images for: ATM-deficient murine thymic T-cell lymphoblastic lymphomas are PTEN-deficient and require AKT signaling for survival
Source: PLoS One. 2024 Dec 5;19(12):e0312864. doi: 10.1371/journal.pone.0312864 (PMC11620668; doi:10.1371/journal.pone.0312864)

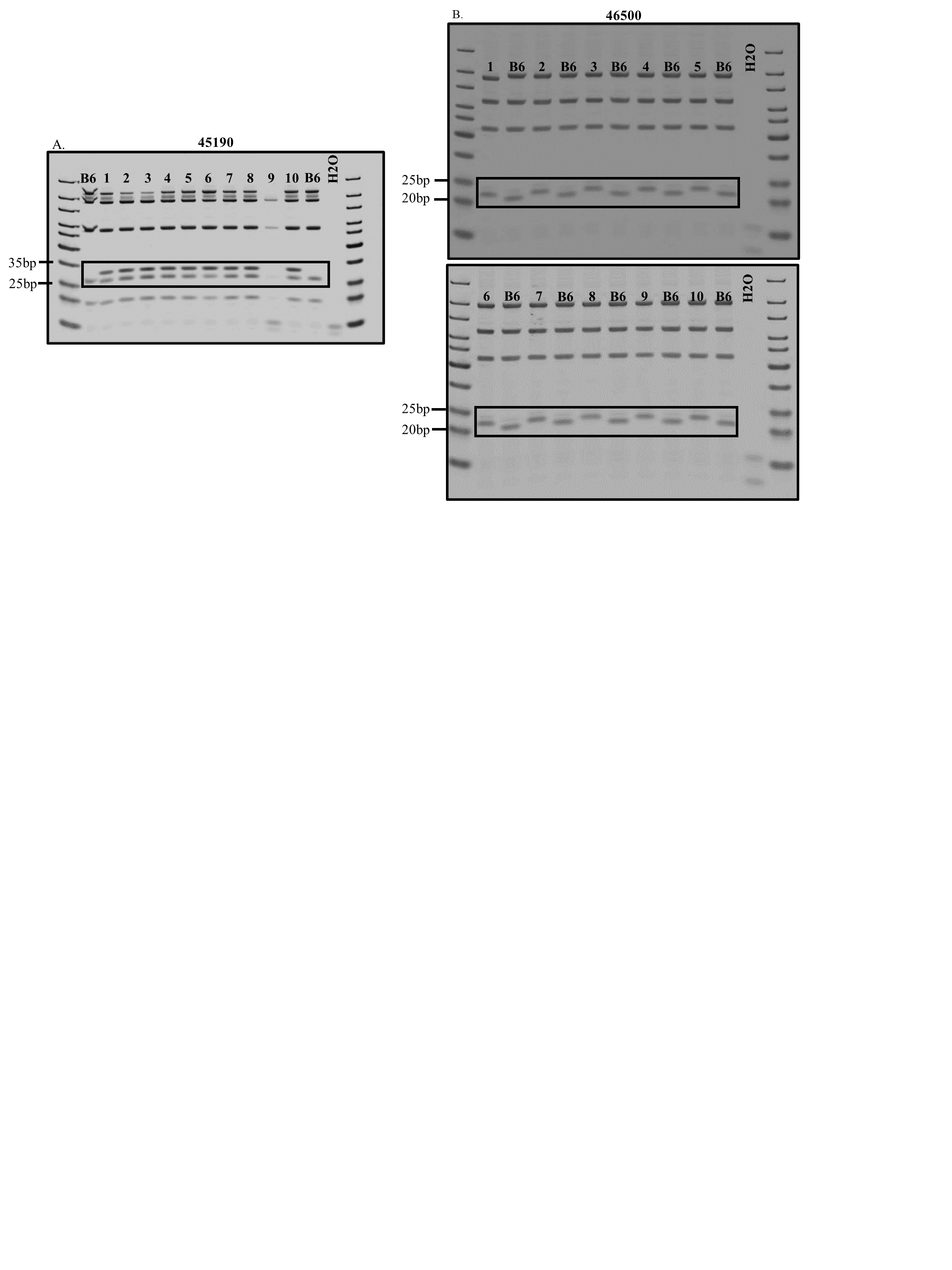

Supplement: S1 Fig — PCR-amplified and restriction enzyme-digested cDNA of tumors 45190 (A) sequenced to have a 32-bp duplication (PstI and BstZI/I-HF enzymes) and 46500 (B) sequenced to have a single-base insertion (RsaI and BfuCI enzymes) were resolved on a 20% polyacrylamide gel. Numbers 1–10 represent technical replicates of each sample and B6 represents wildtype B6 thymus DNA. (TIF) [file pone.0312864.s001.tif]

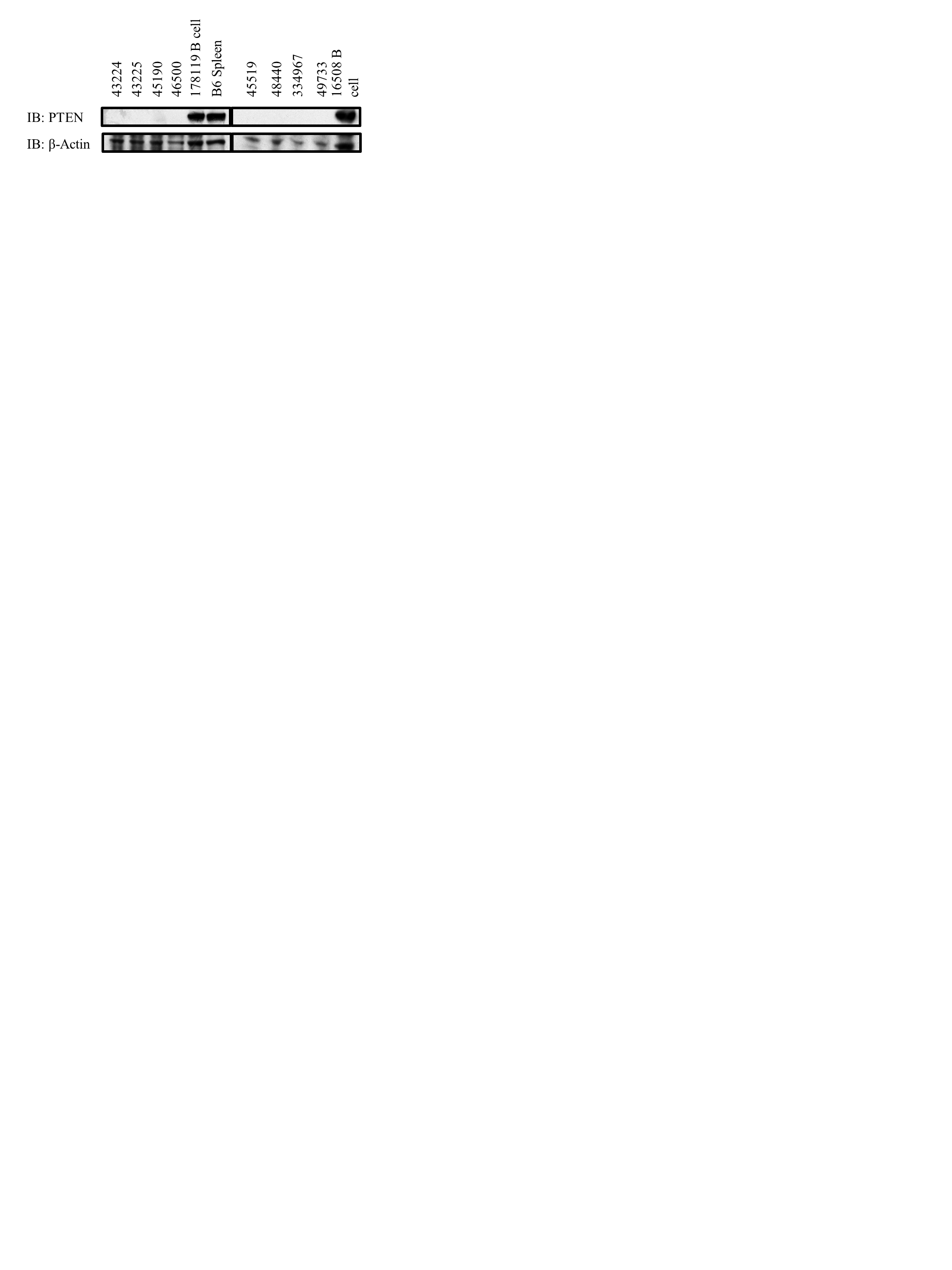

Supplement: S2 Fig — Protein lysates of B6 splenocytes and ATM-deficient T/B cell lymphomas were immunoblotted using antibodies specific for C-terminus of PTEN (Top row) or for β-Actin (bottom row). (TIF) [file pone.0312864.s002.tif]

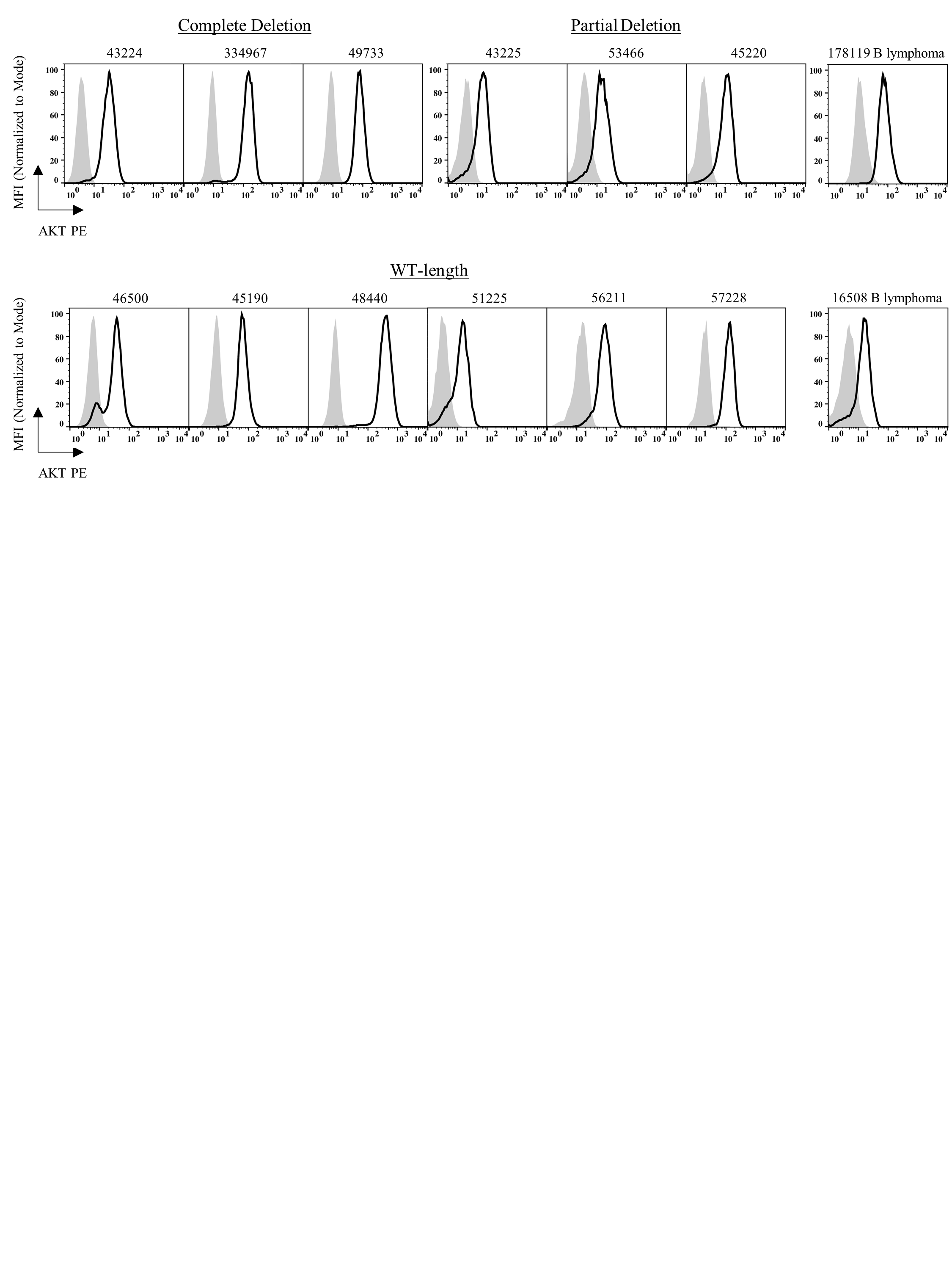

Supplement: S3 Fig — Flow cytometric analysis of ATM-deficient T and B cell lymphomas with PE-conjugated anti-total AKT (black-lined open histogram) or isotype control (gray shaded histogram) antibody grouped by their respective Pten exome profiles. Top row left to right: Staining profiles of ATMKO T-LBL tumors with a complete deletion of the Pten exome (43224, 334967, and 49733), a partial deletion of the exome (43225, 53466, and 45220) and an ATMKO B cell lymphoma (178119). Bottom row left to right: ATMKO T-LBL tumors with a WT-length exome (46500, 45190, 48440, 51225, 56211, and 57228) and an ATMKO B cell lymphoma (16508). Each histogram is representative of three to twelve experiments. (TIF) [file pone.0312864.s003.tif]

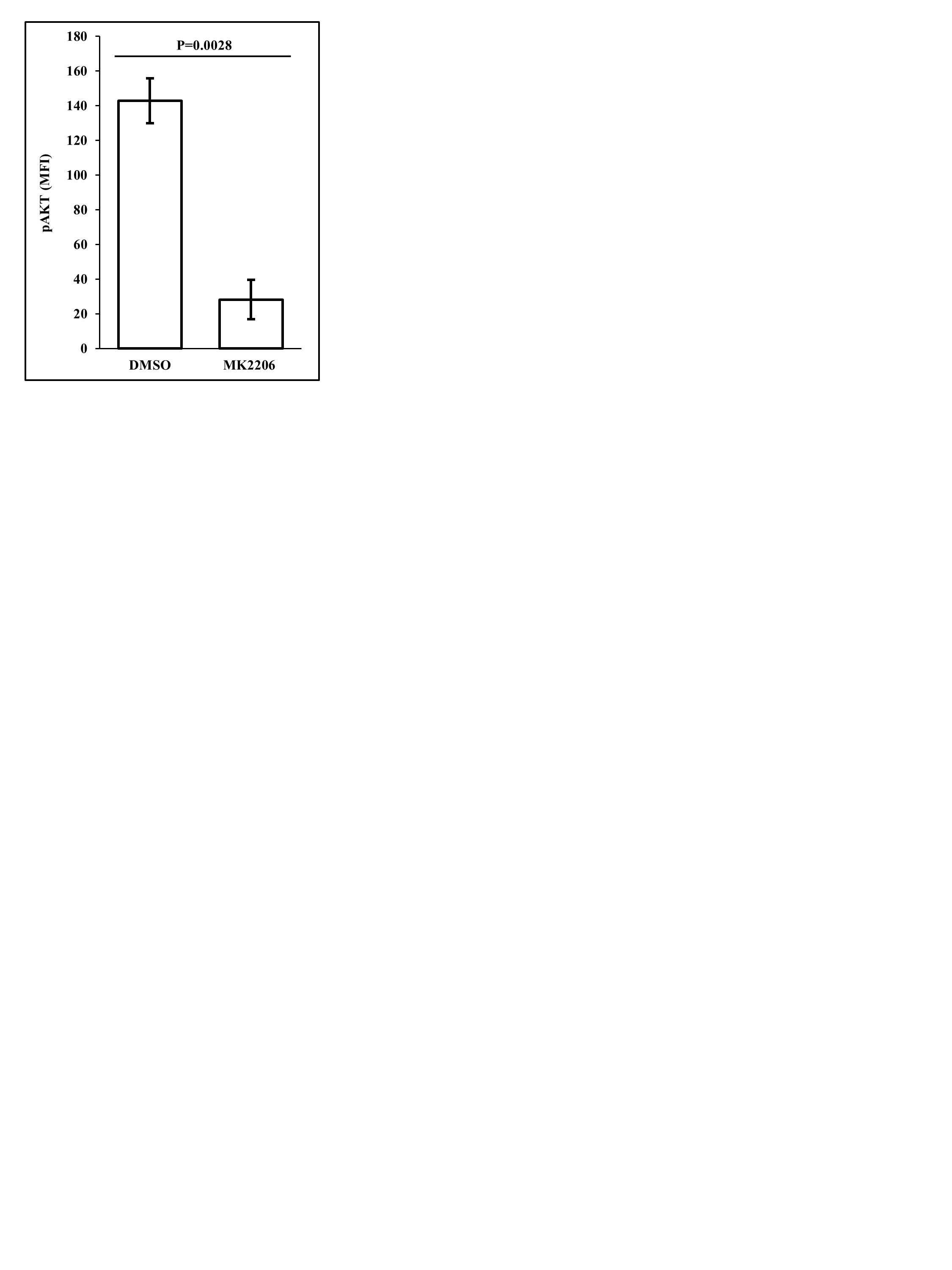

Supplement: S4 Fig — Tumor 43224 mean expression of pAKT (S473) was compared between DMSO control and 200nM MK-2206 after 30 minutes of incubation with +/- SEM across 3 independent experiments. Statistical analysis was performed by using a two sample two-tailed Student’s t-test assuming unequal variances (p values <0.05 were considered statistically significant). (TIF) [file pone.0312864.s004.tif]
