# Supplementary material for: ATM-deficient murine thymic T-cell lymphoblastic lymphomas are PTEN-deficient and require AKT signaling for survival
Source: PLoS One. 2024 Dec 5;19(12):e0312864. doi: 10.1371/journal.pone.0312864 (PMC11620668; doi:10.1371/journal.pone.0312864)
Supplement: S1 Appendix — (DOCX) [file pone.0312864.s005.docx]

**Full Statistical Analysis Summary Report**

**for Figure 4**

| Project objective | To investigate if there is a potential difference between T- and B cell lymphomas in percent inhibition by incubation with varying concentrations of MK2206 |
| --- | --- |
| Date | March 14, 2022 |
| Investigators | Richard Hodes, MD; Joseph An, MD |
| Primary statistician | Hyoyoung Choo-Wosoba, PhD |
| Advisor statistician | Seth Steinberg, PhD |
| Major statistical software | R 4.1.1 |

**Background**

| There are 9 lymphoma cell lines (7 in T-cell and 2 in B-cell) available for this project and no missing values occur across concentrations of MK2206 with the range of 0 to 800 nM. There are varying numbers of technical replicates of percent inhibition in cell viability for each concentration level within each cell line and the averages of those replicate values were used as the outcome measure for this project.  All the analyses are based on the data excluding the concentration of 0 because no variation occurs at zero concentration for MK2206. |
| --- |

**Methods**

| A linear mixed model was considered to investigate the difference in inhibition of cell viability by cell types with increasing levels of MK2206 concentration (fixed effects). In addition to including each of the main effects, the interaction effect between the two measured main effects as well as a random effect *Celli* were included in the model below:  $E\left( Inhibition\% \right)=\mathrm{Concentration}_{MK2206}+Celltype+\mathrm{Concentration}_{MK2206}\times Celltype+{Cell}_{i}$  It should be stated that $\mathrm{Concentration}_{MK2206}$is a continuous variable, as its values represent numerically measured quantities. |
| --- |

**Results**

| Table 1 shows the results for the effect of the major factors (MK2206 concentration and cell type), adjusted for the variations due to different cell lines. There is a likely interaction effect associated with the corresponding coefficient of 0.06 and a small p-value (<0.001). This indicates that the percent inhibition of cell viability increases on average (i.e., population-level inference) at a 0.06 higher rate in T cell lymphomas than that their B cell counterpart as the concentration of MK2206 increases starting from 50 nM. In other words, the percent inhibition on average increases by 0.011 and 0.071 in B- and T cell types, respectively, as the concentration of MK2206 increases by 1 nM. It is also noted that the T cell type tends to have a marginally larger effect on percent inhibition than B cell type, after adjustment for the interaction effect (p-value $\approx$0.05). In addition, estimated variances between and within cell lines are 306.31 and 180.91, respectively (results not shown here). This implies that the variation in the percent inhibitions among cell lines seems larger than that within cell lines.  Figure 1 visually explains the results in Table 1, showing two straight bold lines with different slopes (i.e., 0.011 and 0.071 for B- and T cell types, respectively), reflecting the effect of the interaction term with p=0.0002, and almost completely separated 95% confidence intervals in cell types (green and red areas in Figure 1, reflected by p=0.056). In addition, the regression estimated lines for individual cell lines are shown in Figure 1 (9 broken lines), representing cell line-specific profiles of the percent inhibition deviated from the population-level profiles within cell types (bold solid lines). For an illustration purpose, additional trajectory plots in different styles are also provided in Figure A1 in Appendix A.  Table 1. Population-level estimation summary   \|  \| Estimate \| Standard error \| p-value \| \| --- \| --- \| --- \| --- \| \| Intercept \| 4.14 \| 13.2982 \| 0.757 \| \| $\mathrm{Concentration}_{MK2206}$ \| 0.011 \| 0.0136 \| 0.402 \| \| Celltype^*^ \| 34.45 \| 15.0787 \| 0.056 \| \| $\mathrm{Concentration}_{MK2206}\times Celltype$^*^ \| 0.06 \| 0.0154 \| 0.0002 \|   ^*^ The reference is B cell.  Furthermore, Figure 2 shows a set of boxplots of the percent inhibitions for each level of the concentrations based on raw data, which also agree with the distinctive slopes of two cell types in Figure 1 (bold solid lines). It should be noted that the concentration levels are considered as categorical values so the numeric differences among the levels are not reflected in the x-axis (i.e., the same spacing among boxplots).  Figure 1. The trajectory of percent inhibitions along with MK2206 concentrations and  regression lines^*^  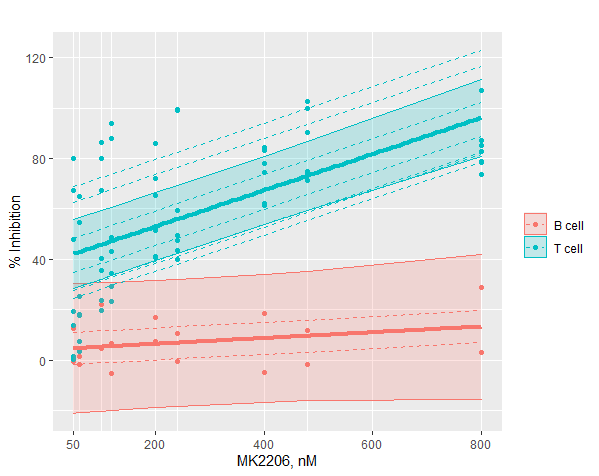  ^*^ The bold solid line for each cell type represents the population-level estimated regression line based on the model; the dashed lines for each cell type represent cell line-specific estimated regression lines based on the model; the area within two solid lines for each cell type refer to 95% confidence intervals of the percent inhibition along with concentrations of MK2207.  Figure 2. Boxplots of the percent inhibitions along with MK2206 concentrations  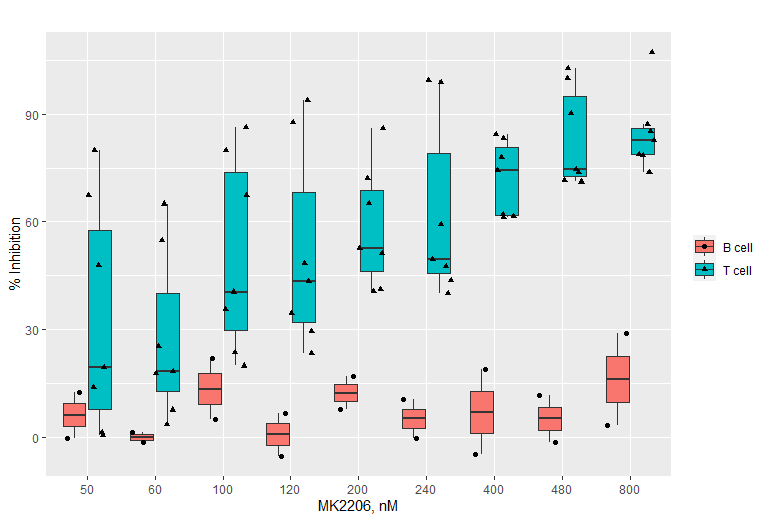 |
| --- | --- | --- | --- | --- | --- | --- | --- | --- | --- | --- | --- | --- | --- | --- | --- | --- | --- | --- | --- | --- |

**Conclusions**

| Based on the model-based and descriptive results above, the effects of MK2206 concentration on percent inhibitions are greater in ATMKO T-LBLs than in ATMKO B cell lymphomas. Overall, there are likely to be different trends in percent inhibition as a function of the concentrations of MK2206 between T- and B cell types (p=.056). |
| --- |

**Appendix A. Additional trajectory plots**

| Figure A1. The percent inhibition profile with MK2206 in different styles^*^   \| 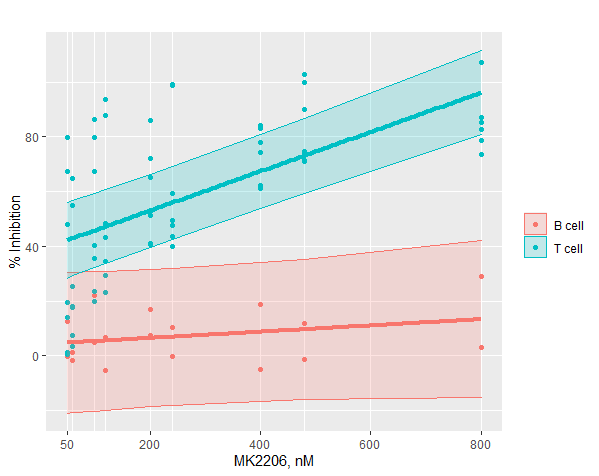 \| 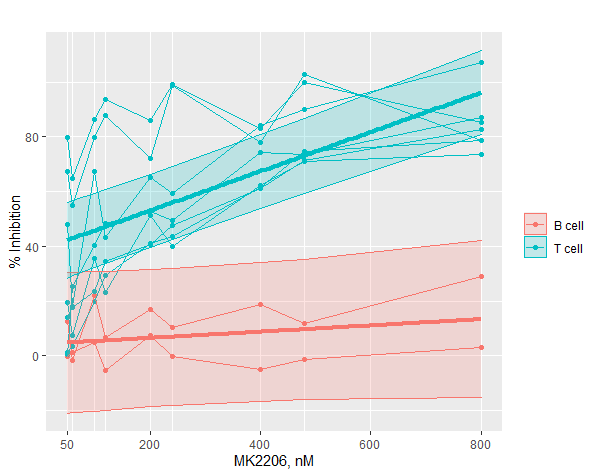 \| \| --- \| --- \| \| (a) \| (b) \| \| 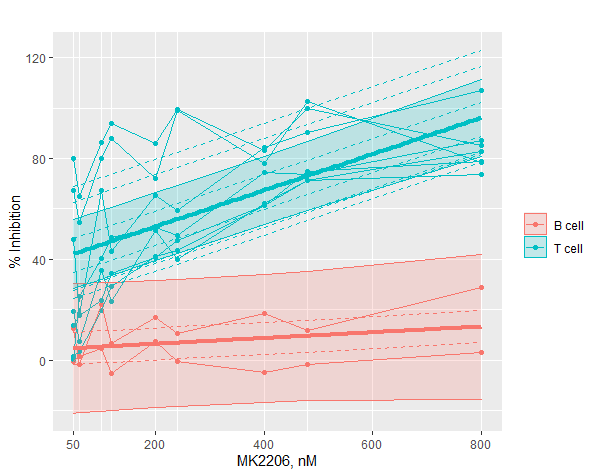 \| ^*^ the solid bold lines: population-level regression line  for each cell type  the colored area: 95% confidence intervals of the  population-level regression lines  within cell types  the broken lines: cell-line specific regression line  the point-connected lines: observed line for each cell  line \| \| (c) \|  \| |
| --- | --- | --- | --- | --- | --- | --- | --- | --- |
